# A Case of Inversio Uteri, Occurring without the Usual Symptoms

**Published:** 1858-10

**Authors:** A. Fisher

**Affiliations:** Chicago, Ill.


ARTICLE V.

### A CASE OF INVERSIO UTERI, OCCURRING WITHOUT THE USUAL SYMPTOMS.

BY A. FISHER, M. D., CHICAGO, ILL.

Mrs. S., aged twenty, bilious and nervous temperament, was taken in labor with her first child, on the morning of April 9, 1858. Her pains were regular, but, about 4 o'clock p. m., in addition to her labor pains, she suddenly began to complain of severe pain in her right side, between the fourth and fifth ribs. Her labor, however, progressed naturally, and she was delivered about 7 o'clock in the evening, with no uncommon symptoms, excepting the pain in her side, which continued without intermission. The cord was rather short, and might possibly have pulled upon the placenta; if so, however, I was not aware of it. The placenta came away at the first pain without any assistance, and everything appeared to be natural, though I did not introduce my hand, as there was no apparent necessity for it.

There was some flooding at the time, but not enough to produce alarm or any signs of syncope. I placed my hand above the pubes, and could feel the uterine tumor; applied cloths, wet in cold water, to insure its permanent contraction and arrest the hemorrhage. The afterpains were about as usual; the pain in the side, however, above-mentioned, between the fourth and fifth ribs, was very severe, and continued so for three or four days, in spite of all our remedies; such as cloths wet with camphorated spirits, sinapisms, Granville's lotion, etc., applied externally, with morphine internally. The only thing she complained of, with the exception of afterpains for a few hours, was the pain in her side, which was so severe and continued so long, that I began to fear that there was some internal lesion, caused by exerting herself whilst in labor.

The third day her bowels were moved by an enema of castor oil and turpentine. The fourth day the pain in her side pretty much subsided, and she was quite comfortable. During this time she had no fever, pain in the back, or tenderness of the bowels; urination was free, and she took some nourishment. The pulse was rather more frequent than natural, and there was little or no secretion of milk, which was attributed to the loss of blood.

From the 13th of April, four days after delivery, she began to improve fast, and complained but little of anything. I visited her daily until the 19th, but gave her no medicine except tonics. There was no flooding, lochia natural, appetite good, bowels regular, and everything appearing right, I discontinued my visits, with a request that they should inform me if she did not do well.

On the 30th, I was called to see the patient's mother, who was sick, and I saw Mrs. S. She appeared to be doing well; said she had been up an hour at a time, and it did not injure her or produce pain in the back. I cautioned her to be careful, as I had been informed that she had had severe hemorrhage and prolapsus uteri for a long time after an abortion, which occurred a year or two previous to her confinement.

On the 7th of May, eighteen days after I had discontinued my visits, I was called to see her again. Her mother said she had been flowing for two or three days, and that she had been up and out to her meals, sung, played on the piano, etc. I attributed her flooding to over exercise before the uterus had recovered its tone. She had no pain in the back, or soreness of the bowels, and the hemorrhage seemed to be her only trouble; for which we applied cold lotions over the uterine region, gave astringents, and enjoined perfect rest. The hemorrhage still continuing, I ordered injection of acetate of lead, which would arrest it for a short time, and then it would return again. As the ordinary means for arresting uterine hemorrhage had been fairly tried, on the 18th of May I made a digital examination, and, to my utter astonishment, found the uterus completely inverted, the fundus resting on the perineum. When and how it took place I cannot imagine. It could not have been at the time the placenta came away, because I felt the uterine tumor above the pubes, and there was no symptom to indicate it at that or any other time, unless the hemorrhage might be regarded as such; but that was not more than is common in many cases where everything is natural.

The next day Professor N. S. Davis was called to consult with me. He questioned the patient and her mother for a long time, very particularly, with regard to her symptoms at the time of her confinement and afterwards, and could not believe that there was inversion, until he made a vaginal examination and found it to be a fact.

We consulted together and concluded that, as the inversion was complete, and the patient very nervous and excitable (so much so that it was with great difficulty that her consent to undergo an examination was obtained), it would be safest and best to encourage her all we could, and not then try to return it; for if we undertook to do it, in the condition she then was, we should surely fail in our attempt, and it might endanger inflammation. We therefore advised astringent injections with nutritious diet, and encouraged her with the hope of a spontaneous replacement, as there were cases of the kind on record well authenticated; intending, all the time, to watch the case and reduce it when she should be in a proper condition.

I visited her daily, or every other day, until the 26th of May, carrying out the above plan, and in two or three weeks she rode a number of miles without inconvenience. She finally concluded to go East and try hydropathic treatment, and left here for that purpose on the 14th of July.

A day or two before she started I saw her husband, and showed him, in the July number of the *American Journal of the Medical Sciences*, reports of two cases of inverted uterus of long standing, said to have been successfully reduced, and urged him to try and persuade her to remain here and let me reduce the inversion in her case, as she was then in a good condition for the operation; telling him at the same time, that I believed it could be reduced, by careful management, without danger, and that no hydropathic treatment could reduce the inversion. But he replied that she had great confidence in cold water treatment, and had determined to try it, and that he would not undertake to dissuade her from doing so. Since she left, as above stated, I have had nothing to do with the case.

The above case is remarkable, from the fact that it clearly proves that the uterus may become inverted without any particular symptoms to warn us of its occurrence. It also shows us that it may take place so gradually that the constitutional symptoms, which usually supervene, may be altogether wanting; so that we would not suspect anything of the kind without a digital examination, which we would not propose, unless the symptoms were such as to make it necessary.

Inverted uterus is of such rare occurrence, that in an active practice of twenty-four years I never before saw but one case, and the symptoms of that were so entirely different from the above, that I will briefly relate it.

The patient had no physician or midwife in attendance before I was called. When I first saw her she was lying on her back convulsing, bathed in a cold perspiration, pulse almost imperceptible; in short, I thought her dying. I immediately gave stimulus with tinc. opii; then, making an examination, I found the placenta, and inverted uterus, between her thighs. The uterine tumor was nearly as large as a child's head, and had passed completely through the vagina. I grasped the tumor with both hands, making steady but firm pressure for a few minutes, until the size was so reduced that I returned it into the vagina, then pressing steadily against the centre, finally replaced it. The uterus remained in its natural position, and convalescence was rapid.

What caused the inversion I could not ascertain. The friends said no one assisted her more than to take away the child, until after its occurrence.

